# Bis[*S*-benzyl 3-(furan-2-yl­methyl­idene)di­thio­carbazato-κ^2^ *N* ^3^,*S*]copper(II): crystal structure and Hirshfeld surface analysis

**DOI:** 10.1107/S2056989019006145

**Published:** 2019-05-14

**Authors:** Enis Nadia Md Yusof, Nazhirah Muhammad Nasri, Thahira B. S. A. Ravoof, Mukesh M. Jotani, Edward R. T. Tiekink

**Affiliations:** a Department of Chemistry, Faculty of Science, Universiti Putra Malaysia, 43400 UPM Serdang, Selangor Darul Ehsan, Malaysia; b Discipline of Chemistry, School of Environmental and Life Sciences, University of Newcastle, University Drive, Callaghan, NSW 2308, Australia; c Department of Chemistry, St. Francis Xavier University, PO Box 5000, Antigonish, NS B2G 2W5, Canada; d Materials Synthesis and Characterization Laboratory, Institute of Advanced Technology, Universiti Putra Malaysia, 43400 UPM Serdang, Selangor Darul Ehsan, Malaysia; e Department of Physics, Bhavan’s Sheth R. A. College of Science, Ahmedabad, Gujarat 380001, India; f Research Centre for Crystalline Materials, School of Science and Technology, Sunway University, 47500 Bandar Sunway, Selangor Darul Ehsan, Malaysia

**Keywords:** crystal structure, copper, di­thio­carbazato, Hirshfeld surface analysis

## Abstract

The title Cu^II^ complex, [Cu(C_13_H_11_N_2_OS_2_)_2_], features a *trans*-N_2_S_2_ donor set as a result of the Cu^II^ atom being located on a crystallographic centre of inversion and being coordinated by thiol­ate-S and imine-N atoms derived from two di­thio­carbazate anions. The resulting geometry is distorted square-planar. In the crystal, π(chelate ring)–π(fur­yl) [inter-centroid separation = 3.6950 (14) Å and angle of inclination = 5.33 (13)°] and phenyl-C—H⋯π(phen­yl) inter­actions sustain supra­molecular layers lying parallel to (02). The most prominent inter­actions between layers, as confirmed by an analysis of the calculated Hirshfeld surface, are phenyl-H⋯H(phen­yl) contacts. Indications for Cu⋯*Cg*(fur­yl) contacts (Cu⋯*Cg* = 3.74 Å) were also found. Inter­action energy calculations suggest the contacts between mol­ecules are largely dispersive in nature.

## Abstract 2

The title Cu^II^ di­thio­carbazate complex features a square-planar *trans*-N_2_S_2_ donor set for the metal atom (site symmetry 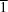). Supra­molecular layers parallel to (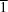
02) are found in the crystal, being sustained by π–π(fur­yl) and C—H⋯π inter­actions.

## Chemical context   

Di­thio­carbaza­tes, derived from sulfur–nitro­gen donor ligands were first reviewed in the 1970s (Ali & Livingstone, 1974 ▸). These Schiff base mol­ecules are readily prepared from the reaction of primary amines with aldehydes or ketones and are potentially multidentate ligands for metals (Ali *et al.*, 2005 ▸; Mokhtaruddin *et al.*, 2017 ▸). Schiff bases display significant biological and pharmacological activities that can be tuned by incorporating different types of substituents through the condensation reaction (How *et al.*, 2008 ▸; Low *et al.*, 2016 ▸). Transition-metal complexes containing Schiff base ligands have also been intensively studied because of their simple routes of synthesis, the variety of their structural geometries and, particularly pertinent, as small chemical changes often produce wide variations in their bioactivities (Mirza *et al.*, 2014 ▸; Zangrando *et al.*, 2015 ▸; Lima *et al.*, 2018 ▸). Recently, a copper(II) di­thio­carbazate complex containing a Schiff base derived from *S*-hexyl­dithio­carbazate and 4-methyl­benzaldehyde was reported to have excellent anti-bacterial activity against *Escherichia coli* (Zangrando *et al.*, 2017 ▸). More recently, investigators have reported the potent biological activity of a copper(II) complex that contained a tridentate Schiff base derived from *S-*benzyl­dithio­carbazate and 2-hy­droxy-5-(phenyl­diazen­yl)benzaldehyde against a human cervical cancer line (HeLa) (Kongot *et al.*, 2019 ▸). The copper(II) complex had comparable biological activities as the well-known anti-cancer drug cisplatin against the tested cells (Kongot *et al.*, 2019 ▸). As part of on-going studies in the structural chemistry and potential bioactivity of copper(II) complexes containing di­thio­carbazate Schiff base ligands, herein the synthesis of the title copper(II) complex, (I), its single crystal X-ray diffraction analysis and a detailed study of supra­molecular association by an analysis of calculated Hirshfeld surfaces and computation chemistry are described.
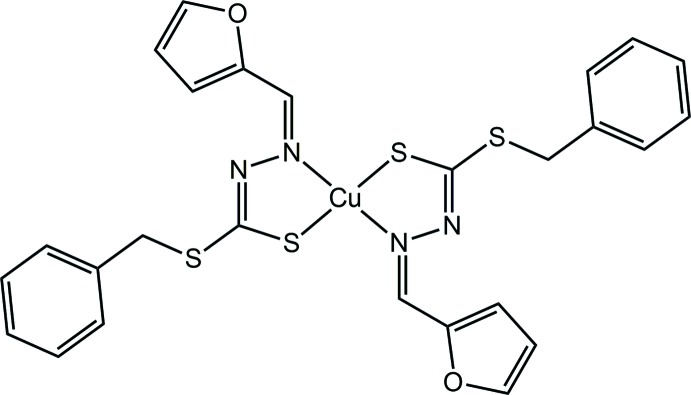


## Structural commentary   

**Figure 1:**
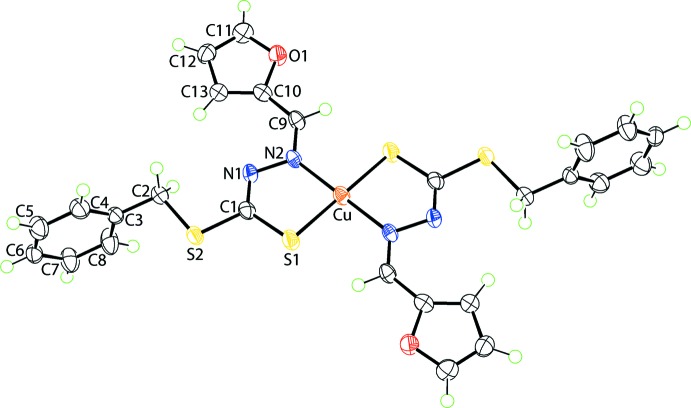
The mol­ecular structure of (I) showing the atom-labelling scheme and displacement ellipsoids at the 70% probability level. Unlabelled atoms are related by the symmetry operation 1 − *x*, 1 − *y*, 1 − *z*.

The mol­ecular structure of (I), Fig. 1 ▸, has the Cu^II^ atom located on a crystallographic centre of inversion and coordin­ated by two chelating di­thio­carbazate anions, each *via* the thiol­ate-S and imine-N atoms (Table 1 ▸). The resulting *trans*-N_2_S_2_ donor set defines a distorted square-planar geometry: the major distortion from the ideal angles subtended at the copper atom is the acute S1—Cu—N2 chelate angle of 85.83 (6)°. The conformation about the endocylic imine bond is *Z*, as a result of chelation, whereas the exocyclic imine bond has an *E* conformation.

The bidentate mode of the coordination of the di­thio­carbazate ligand leads to the formation of five-membered CuN_2_CS chelate rings. While the r.m.s. deviation for the five atoms is relatively small at 0.0453 Å, suggesting a near planar ring, a better description for the conformation is that of an envelope with the copper atom being the flap atom. In this description, the r.m.s. deviation of the S1, N1, N2 and N3 atoms of the ring is 0.0002 Å, with the Cu atom lying 0.199 (3) Å out of the plane. The dihedral angle between the best plane through the chelate ring and the 2-furyl ring is 5.33 (18)° indicating an essentially co-planar relationship. By contrast, the dihedral between the chelate and phenyl rings is 86.75 (7)°, indicative of an orthogonal relationship. Finally, the dihedral angle between the peripheral organic rings is 81.42 (9)°.

The structure of the acid form of the anion in (I) is available for comparison (Shan *et al.*, 2008 ▸). Referring to the data in Table 1 ▸, significant changes in key bond lengths have occurred upon deprotonation and coordination of the mol­ecule to Cu^II^ in (I). Thus, the C1—S1 [1.669 (2) Å for the acid], N1—N2 [1.381 (2) Å] and C9—N2 [1.280 (3) Å] bond lengths have all elongated in (I), Table 1 ▸, while the C1—N1 bond length has shortened [1.336 (3) Å]. Significant changes in the angles subtended at the quaternary C1 atom are also noted, in particular for the S1—C1—S2 angle which has narrowed by *ca* 10° in (I) from 124.76 (12)° in the acid with concomitant widening of the S2—C1–N1 angle by *ca* 5°, changes consistent with the reorganization of π-electron density from the C1—S1 to C1—N1 bonds in (I).

## Supra­molecular features   

**Figure 2:**
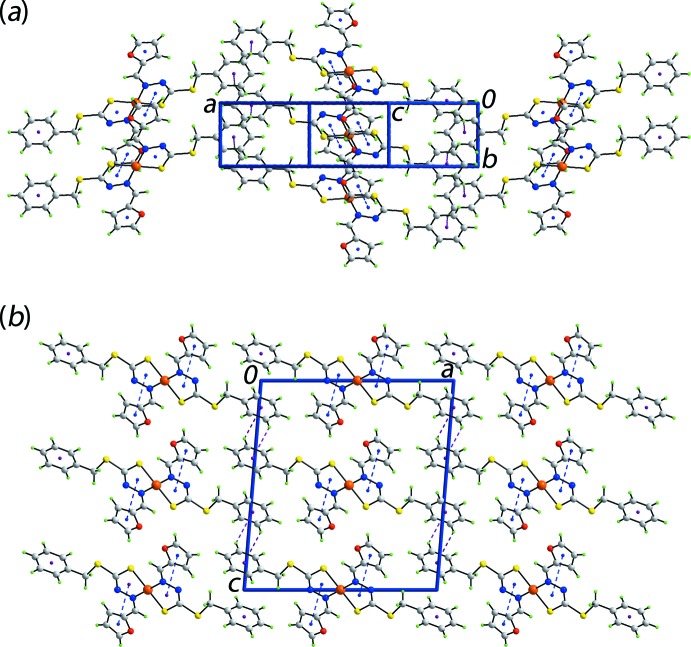
Mol­ecular packing in (I): (*a*) a view of the supra­molecular layer sustained by π(chelate ring)–π(fur­yl) and phenyl-C—H⋯π(phen­yl) inter­actions shown as blue and purple dashed lines, respectively, and (*b*) a view of the unit-cell contents shown in projection down the *b* axis highlighting the stacking of layers.

The most prominent feature of the mol­ecular packing is the formation of supra­molecular layers lying parallel to (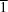
02), Fig. 2 ▸(*a*). The association between mol­ecules is of the type π(chelate ring)–π(fur­yl) whereby the inter-centroid *Cg*(Cu,S1,N1,N2,C1)–*Cg*(O1,C10–C13)^i^ separation is 3.6950 (14) Å with angle of inclination = 5.33 (13)°; symmetry operation (i) *x*, −1 + *y*, *z*. Such π–π inter­actions between chelate rings and aromatic rings are well documented in the literature, especially for sterically unencumbered square-planar complexes and can impart significant energies of stabilization to the mol­ecular packing (Malenov *et al.* 2017 ▸; Tiekink, 2017 ▸). In the present case, these inter­actions link mol­ecules along the *b*-axis direction. Links between the chains to form layers are of the type phenyl-C—H⋯π(phen­yl), Table 2 ▸. A view of the unit-cell contents is shown in Fig. 2 ▸(*b*). Details of the weak inter­molecular contacts connecting layers are given in the analysis of the calculated Hirshfeld surfaces below.

## Analysis of the Hirshfeld surfaces   

**Figure 3:**
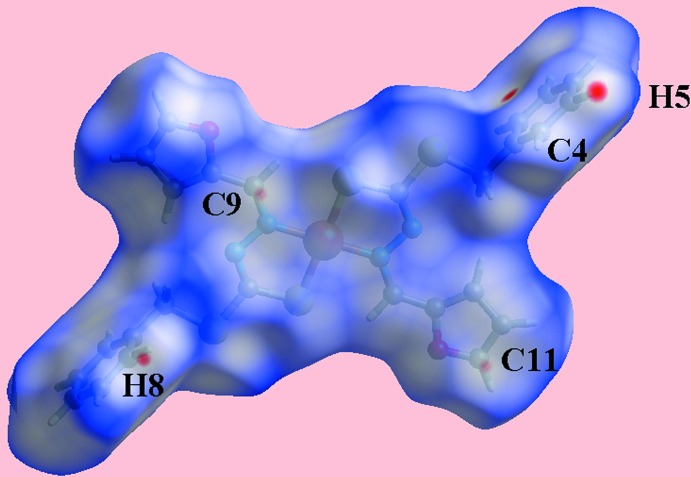
A view of the Hirshfeld surface for (I) mapped over *d*_norm_ in the range −0.080 to +1.213 arbitrary units.

**Figure 4:**
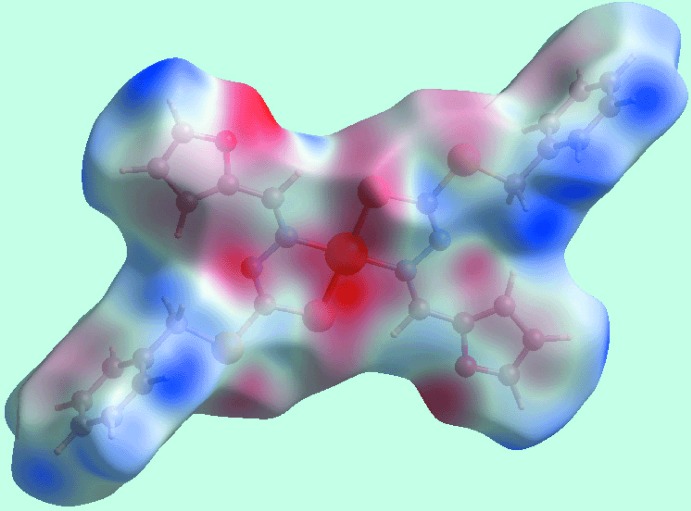
A view of the Hirshfeld surface for (I) mapped over the electrostatic potential in the range −0.036 to + 0.034 atomic units.

**Figure 5:**
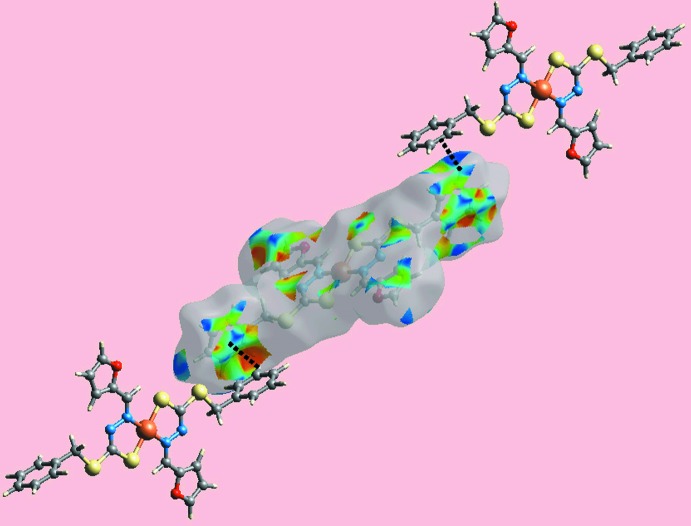
A view of the Hirshfeld surface with the shape-index property highlighting C—H⋯π/π⋯H—C contacts by black dotted lines.

The analysis of the Hirshfeld surfaces calculated for (I) was conducted as per literature precedents (Tan *et al.*, 2019 ▸) employing *Crystal Explorer* (Turner *et al.*, 2017 ▸). The assumption of the inter­molecular C—H⋯π contact in the crystal of (I) is justified through the diminutive red spots near the phenyl-C4 and H5 atoms on the Hirshfeld surfaces mapped over *d*_norm_ in Fig. 3 ▸. The short inter­atomic H⋯H contact, involving phenyl H8 atoms and occurring between layers, and the C⋯C contact, between the methyl­ene-C9 and furyl-C11 atoms, are also evident as the faint-red spots near the respective atoms in Fig. 3 ▸. On the Hirshfeld surfaces mapped over electrostatic potential in Fig. 4 ▸, the donors and acceptors of inter­molecular C—H⋯π contacts, Table 2 ▸, are viewed as blue bumps and light-red concave regions, respectively. Also, the short inter­atomic S⋯H/H⋯S contacts, which are electrostatic in nature, Table 3 ▸, show red and blue regions about the respective atoms. The environment around a reference mol­ecule within the Hirshfeld surface mapped with the shape-index property is illustrated in Fig. 5 ▸, and highlights the C—H⋯π/π⋯H—C contacts.

**Figure 6:**
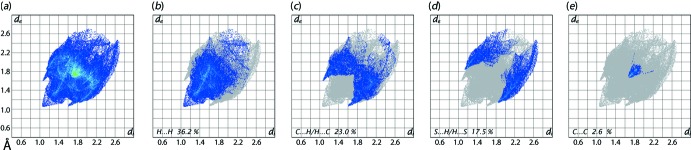
(*a*) The full two-dimensional fingerprint plot for (I) and fingerprint plots delineated into (*b*) H⋯H, (*c*) C⋯H/H⋯C, (*d*) S⋯H/H⋯S and (*e*) C⋯C contacts.

The overall two-dimensional fingerprint plot, Fig. 6 ▸(*a*), and those delineated into H⋯H, C⋯H/H⋯C, S⋯H/H⋯S and C⋯C contacts are illustrated in Fig. 6 ▸(*b*)–(*e*), respectively; the percentage contribution from all the identified inter­atomic contacts to the Hirshfeld surface are summarized qu­anti­tatively in Table 4 ▸.

The conical tip appearing at *d*_e_ + *d*_i_ ∼2.1 Å in the fingerprint plot delineated into H⋯H contacts in Fig. 6 ▸(*b*), represents the short inter-layer H⋯H contact involving phenyl-H8 atoms, Table 3 ▸. The presence of the C—H⋯π inter­action is evident through the short inter­atomic C⋯H/H⋯C contact characterized as the pair of forceps-like tips at *d*_e_ + *d*_i_ ∼2.7 Å in the respective delineated fingerprint plot of Fig. 6 ▸(*c*) and Table 3 ▸. In the fingerprint plot delineated into S⋯H/H⋯S contacts, Fig. 6 ▸(*d*), the short inter­atomic contact involving the S-benzyl atoms, Table 3 ▸, appear as the pair of forceps-like tips at *d*_e_ + *d*_i_ < 3.0 Å, *i.e*. at the sum of van der Waals radii. The distribution of points in the fingerprint plot delineated into C⋯C contacts, Fig. 6 ▸(*e*), forming triangular tip at *d*_e_ + *d*_i_ ∼3.3 Å is due to the presence of such short inter­atomic contacts summarized in Table 3 ▸. The presence of inter­molecular π–π stacking between chelate and furyl rings results in the small but significant percentage contribution from the participating atoms, as listed in Table 4 ▸. The small contributions from the other remaining inter­atomic contacts summarized in Table 4 ▸ have a negligible effect on the packing.

## Computational chemistry   

**Figure 7:**
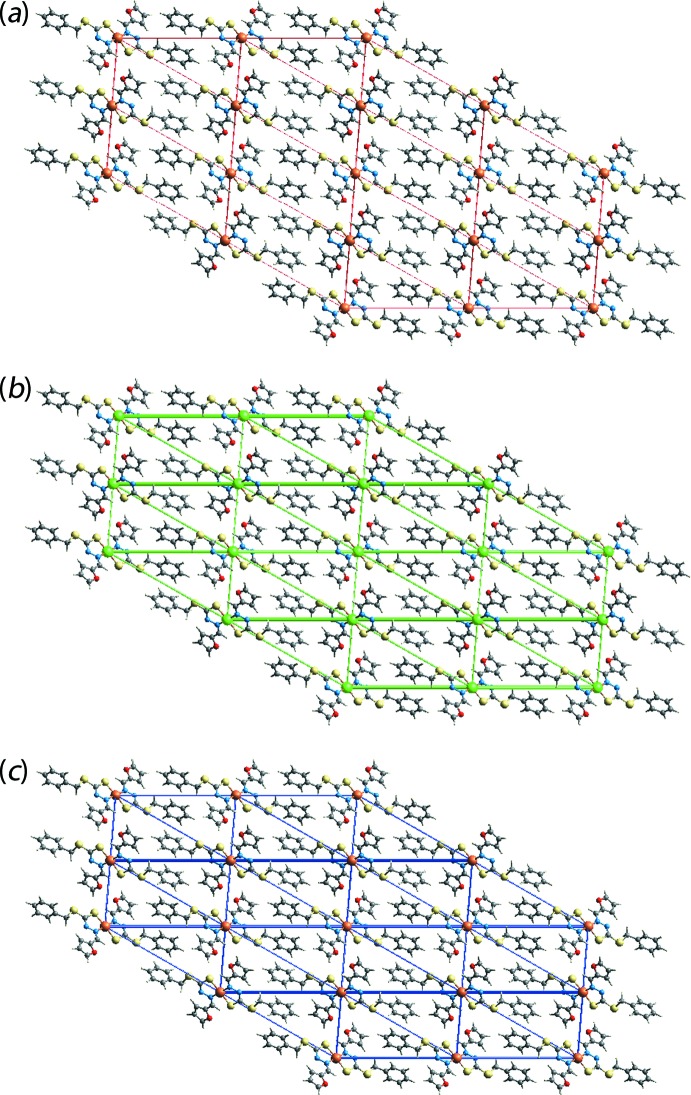
The energy frameworks viewed down the *b*-axis direction comprising (*a*) electrostatic potential force, (*b*) dispersion force and (*c*) total energy for a cluster about a reference mol­ecule of (I). The energy frameworks were adjusted to the same scale factor of 50 with a cut-off value of 3 kJ mol^−1^ within 2 × 2 × 2 unit cells.

Utilizing *Crystal Explorer* (Turner *et al.*, 2017 ▸), the pairwise inter­action energies between the mol­ecules within the crystal were calculated by summing up four energy component, namely electrostatic (*E*_ele_), polarization (*E*_pol_), dispersion (*E*_dis_) and exchange-repulsion (*E*_rep_). The energies were obtained using the wave function calculated at the HF/STO-3G level theory. The strength and nature of the inter­molecular inter­actions are summarized qu­anti­tatively in Table 5 ▸. From the inter­action energies calculated between the reference mol­ecule and the symmetry-related mol­ecule at *x*, −1 + *y*, *z* in Table 5 ▸, it is observed that the greatest energy value is due to the combined influence of Cu⋯furyl [Cu⋯*Cg*(fur­yl) = 3.74 Å], π(chelate)–π(fur­yl), C⋯C and S⋯H/H⋯S inter­actions. Among these inter­actions, the short inter­atomic S⋯H/H⋯S contact contributes to the electrostatic component while the others to the dispersion component of the energies. Even though the inter-centroid distance between symmetry-related phenyl (C3–C8) rings are greater than 4.0 Å [*Cg*⋯*Cg* ^i^ = 4.3102 (17) Å; (i) − *x*, 2 − *y*, 1 − *z*] and the inter­atomic S⋯H distance is greater than sum of their van der Waal radii (S1⋯H11^ii^ = 3.11 Å; *x*, 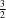 − *y*, −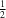 + *z*), they possess greater inter­action energies compared to inter­molecular phenyl-C—H⋯π(phen­yl) and short inter­atomic H⋯H contacts, as summarized in Table 5 ▸. The magnitudes of the inter­molecular energies are represented graphically in the energy frameworks down the *b*-axis direction in Fig. 7 ▸. Here, the supra­molecular architecture of crystals is viewed through the cylinders joining the centroids of mol­ecular pairs by using red, green and blue colour codes for the components *E*_ele_, *E*_disp_ and *E*_tot_, respectively; the radius of the cylinder is proportional to the magnitude of inter­action energy. It is clearly evident from the energy frameworks shown in Fig. 7 ▸ that the major contribution to the inter­molecular inter­actions is from the dispersion energy component in the absence of conventional hydrogen bonds in the crystal.

## Database survey   

The Cambridge Structural Database (Groom *et al.*, 2016 ▸) contains just about 100 structures with the basic core found in (I). Manual sorting to identify ligands without additional donors as in (I), *e.g*. substituents carrying pyridyl or phenoxide, neutral mol­ecules only and non-solvated structures yielded 24 analogues to (I) with deposited atomic coordinates. Eleven of these structures adopt the *trans*-N_2_S_2_ square-planar geom­etry as in (I), while the remaining 13 structures adopt a flattened tetra­hedral coordination geometry. The structural di­versity exhibited by these complexes is emphasized by the bi­nuclear species [Cu{SCS[(CH_2_)_5_Me]=NN=CC_6_H_4_OMe-4}_2_]_2_ arising from inter­molecular Cu⋯S inter­actions between centrosymmetrically related *trans*-N_2_S_2_ square-planar geometries (Begum *et al.*, 2017 ▸).

## Synthesis and crystallization   

**Synthesis of the 2-furaldehyde Schiff base of** ***S*** **-benzyl­dithio­carbazate:** *S*-Benzyl­dithio­carbazate (SBDTC) was synthesized following a procedure adapted from a previous report (Tarafder *et al.*, 2001 ▸). The Schiff base was synthesized using a procedure adapted from the literature (Yusof *et al.*, 2015 ▸) by reacting SBDTC (3.96 g, 0.02 mol) and an equimolar amount of 2-furaldehyde (1.92 g, 0.02 mmol) in hot ethanol (20 ml). The mixture was then heated until the volume reduced to half, followed by stirring under room temperature until a precipitate had formed. The resulting Schiff base was then washed with ice-cold ethanol, recrystallized from ethanol solution and dried over silica gel. Colour: Yellow. Yield 94%, m.p. 447–449 K. Elemental analysis: Calculated for C_13_H_12_N_2_OS_2_: C, 56.49; H, 4.38; N, 10.14. Found; C, 56.64; H, 4.21; N, 9.64. FTIR (ATR, cm^−1^): 3089 (*w*) ν(N—H), 1609 (*m*) ν(C=N), 1016 (*s*) ν(N—N), 763 (*s*), ν(C=S).

Synthesis of (I): The Schiff base synthesized above (0.55 g, 0.002 mol) was dissolved in hot ethanol (50 ml) and added to copper(II) acetate monohydrate (0.20 g, 0.001 mol) in an ethano­lic solution (30 ml). The mixture was heated until the volume of the solution reduced to half. Precipitation occurred once the mixture had cooled to room temperature. The precipitate was filtered and dried over silica gel. The title complex was recrystallized from methanol solution as dark-brown prisms in 91% yield. M.p. 456–458 K. Elemental analysis: Calculated for C_26_H_22_CuN_4_O_2_S_4_: C, 50.84; H, 3.61; N, 9.12; Cu, 10.34. Found; C, 50.49; H, 3.45; N, 8.77; Cu, 10.81. FTIR (ATR, cm^−1^): 1593 (*m*), ν(C=N), 964 (*s*), ν(N—N), 760 (*s*), ν(C—S).

## Refinement   

Crystal data, data collection and structure refinement details are summarized in Table 6 ▸. The carbon-bound H atoms were placed in calculated positions (C—H = 0.95–0.99 Å) and were included in the refinement in the riding-model approximation, with *U*_iso_(H) set to 1.2*U*_eq_(C).

## Supplementary Material

Crystal structure: contains datablock(s) I, global. DOI: 10.1107/S2056989019006145/hb7822sup1.cif

Structure factors: contains datablock(s) I. DOI: 10.1107/S2056989019006145/hb7822Isup2.hkl

CCDC reference: 1913482

Additional supporting information:  crystallographic information; 3D view; checkCIF report

## Figures and Tables

**Table 1:** Selected geometric parameters (Å, °)

Cu—S1	2.1845 (7)	N1—N2	1.409 (3)
Cu—N2	1.923 (2)	C1—N1	1.286 (3)
C1—S1	1.720 (3)	C9—N2	1.300 (3)
C1—S2	1.753 (2)		
			
S1—Cu—N2	85.83 (6)	S1—C1—N1	125.08 (19)
S1^i^—Cu—N2	94.18 (6)	S2—C1—N1	119.9 (2)
S1—C1—S2	115.03 (15)		

Symmetry code: (i) 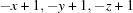.

**Table 2:** Hydrogen-bond geometry (Å, °) *Cg*1 is the centroid of the (C3–C8) ring.

*D*—H⋯*A*	*D*—H	H⋯*A*	*D*⋯*A*	*D*—H⋯*A*
C5—H5⋯*Cg*1^ii^	0.95	2.96	3.646 (3)	131

Symmetry code: (ii) 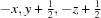.

**Table 3:** Summary of short inter­atomic contacts (Å) in (I)

Contact	Distance	Symmetry operation
H8⋯H8	2.11	−*x*, 1 − *y*, 1 − *z*
H5⋯C4	2.66	−*x*, 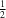 + *y*, 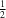 − *z*
H2*B*⋯S2	2.97	*x*, 1 + *y*, *z*
C9⋯C11	3.364 (4)	*x*, −1 + *y*, *z*

Notes: (*a*) The inter­atomic distances are calculated in *Crystal Explorer* (Turner *et al.*, 2017 ▸) whereby the *X*—H bond lengths are adjusted to their neutron values.

**Table 4:** Percentage contributions of inter­atomic contacts to the Hirshfeld surface for (I)

Contact	Percentage contribution
H⋯H	36.2
C⋯H/H⋯C	23.0
S⋯H/H⋯S	17.5
O⋯H/H⋯O	5.1
C⋯N/N⋯C	3.3
S⋯O/O⋯S	2.9
N⋯H/H⋯N	2.8
Cu⋯C/C⋯Cu	2.7
C⋯C	2.6
C⋯S/S⋯C	1.3
N⋯S/S⋯N	1.2
O⋯O	0.5
N⋯O/O⋯N	0.3
N⋯N	0.3
Cu⋯N/N⋯Cu	0.2
Cu⋯H/H⋯Cu	0.1
Cu⋯O/O⋯Cu	0.1

**Table 5:** Summary of inter­action energies (kJ mol^−1^) calculated for (I)

Contact	*R* (Å)	*E*_ele_	*E*_pol_	*E*_dis_	*E*_rep_	*E*_tot_
Cu⋯*Cg*(fur­yl)^i^ +						
*Cg*(chelate)⋯*Cg*(fur­yl)^i^ +						
C9⋯C11^i^ +						
S2⋯H2*B* ^i^	5.02	−23.2	−9.4	−154.4	97.6	−89.7
*Cg*(phen­yl)⋯*Cg*(phen­yl)^ii^	16.15	−6.3	−3.3	−50.9	28.3	−31.5
S1⋯H11^iii^	11.25	−12.0	−2.6	−10.6	5.2	−19.2
C5—H5⋯*Cg*(phen­yl)^iv^	17.06	−6.2	−2.1	−20.6	13.8	−15.1
H8⋯H8^v^	15.35	0.7	−0.9	−15.6	7.9	−7.5

Notes: Symmetry operations: (i) *x*, −1 + *y*, *z*; (ii) −*x*, 2 − *y*, 1 − *z*; (iii) *x*, 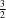 − *y*, 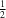 + *z*; (iv) −*x*, 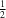 + *y*, 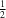 − *z*; (v) −*x*, 1 − *y*, 1 − *z*.

**Table 6:** Experimental details

Crystal data	
Chemical formula	[Cu(C_13_H_11_N_2_OS_2_)_2_]
*M*_r_	614.25
Crystal system, space group	Monoclinic, *P*2_1_/*c*
Temperature (K)	100
*a*, *b*, *c* (Å)	15.3515 (7), 5.0151 (3), 16.7186 (8)
β (°)	94.618 (4)
*V* (Å^3^)	1282.98 (11)
*Z*	2
Radiation type	Mo *K*α
μ (mm^−1^)	1.21
Crystal size (mm)	0.30 × 0.20 × 0.10

Data collection	
Diffractometer	Agilent Xcalibur Eos Gemini
Absorption correction	Multi-scan (*CrysAlis PRO*; Agilent, 2011 ▸)
*T*_min_, *T*_max_	0.744, 1.000
No. of measured, independent and observed [*I* > 2σ(*I*)] reflections	5864, 2898, 2382
*R*_int_	0.027
(sin θ/λ)_max_ (Å^−1^)	0.677

Refinement	
*R*[*F* ^2^ > 2σ(*F* ^2^)], *wR*(*F* ^2^), *S*	0.040, 0.111, 1.04
No. of reflections	2898
No. of parameters	169
H-atom treatment	H-atom parameters constrained
Δρ_max_, Δρ_min_ (e Å^−3^)	0.49, −0.61

Computer programs: *CrysAlis PRO* (Agilent, 2011 ▸), *SHELXS97* (Sheldrick, 2008 ▸), *SHELXL2014* (Sheldrick, 2015 ▸), *ORTEP-3 for Windows* (Farrugia, 2012 ▸), *DIAMOND* (Brandenburg, 2006 ▸) and *publCIF* (Westrip, 2010 ▸).

## supplementary crystallographic information

### Crystal data

**Table:**

[Cu(C_13_H_11_N_2_OS_2_)_2_]	*F*(000) = 630
*M**_r_* = 614.25	*D*_x_ = 1.590 Mg m^−^^3^
Monoclinic, *P*2_1_/*c*	Mo *K*α radiation, λ = 0.71073 Å
*a* = 15.3515 (7) Å	Cell parameters from 1956 reflections
*b* = 5.0151 (3) Å	θ = 2.4–28.7°
*c* = 16.7186 (8) Å	µ = 1.21 mm^−^^1^
β = 94.618 (4)°	*T* = 100 K
*V* = 1282.98 (11) Å^3^	Prism, dark-brown
*Z* = 2	0.30 × 0.20 × 0.10 mm

### Data collection

**Table:**

Agilent Xcalibur Eos Gemini diffractometer	2898 independent reflections
Radiation source: fine-focus sealed X-ray tube, Enhance (Mo) X-ray Source	2382 reflections with *I* > 2σ(*I*)
Graphite monochromator	*R*_int_ = 0.027
Detector resolution: 16.1952 pixels mm^-1^	θ_max_ = 28.8°, θ_min_ = 2.4°
ω scans	*h* = −19→18
Absorption correction: multi-scan (CrysAlis PRO; Agilent, 2011)	*k* = −5→6
*T*_min_ = 0.744, *T*_max_ = 1.000	*l* = −22→20
5864 measured reflections	

### Refinement

**Table:**

Refinement on *F*^2^	Primary atom site location: structure-invariant direct methods
Least-squares matrix: full	Secondary atom site location: difference Fourier map
*R*[*F*^2^ > 2σ(*F*^2^)] = 0.040	Hydrogen site location: inferred from neighbouring sites
*wR*(*F*^2^) = 0.111	H-atom parameters constrained
*S* = 1.03	*w* = 1/[σ^2^(*F*_o_^2^) + (0.0542*P*)^2^ + 1.1451*P*] where *P* = (*F*_o_^2^ + 2*F*_c_^2^)/3
2898 reflections	(Δ/σ)_max_ < 0.001
169 parameters	Δρ_max_ = 0.49 e Å^−^^3^
0 restraints	Δρ_min_ = −0.61 e Å^−^^3^

### Special details

**Table:**

Geometry. All esds (except the esd in the dihedral angle between two l.s. planes) are estimated using the full covariance matrix. The cell esds are taken into account individually in the estimation of esds in distances, angles and torsion angles; correlations between esds in cell parameters are only used when they are defined by crystal symmetry. An approximate (isotropic) treatment of cell esds is used for estimating esds involving l.s. planes.

### Fractional atomic coordinates and isotropic or equivalent isotropic displacement parameters (Å^2^)

**Table:**

	*x*	*y*	*z*	*U*_iso_*/*U*_eq_	
Cu	0.5000	0.5000	0.5000	0.01942 (15)	
S1	0.40280 (4)	0.46830 (15)	0.39759 (4)	0.02392 (18)	
S2	0.22892 (4)	0.71156 (14)	0.37681 (4)	0.02121 (18)	
O1	0.42829 (12)	1.2573 (4)	0.69781 (11)	0.0240 (4)	
N1	0.34226 (13)	0.8207 (4)	0.49969 (13)	0.0186 (5)	
N2	0.42578 (13)	0.7729 (4)	0.53831 (13)	0.0168 (5)	
C1	0.32893 (16)	0.6845 (5)	0.43470 (16)	0.0185 (5)	
C2	0.17122 (17)	0.9561 (6)	0.43348 (17)	0.0221 (6)	
H2A	0.1756	0.9075	0.4911	0.027*	
H2B	0.1976	1.1347	0.4281	0.027*	
C3	0.07652 (17)	0.9609 (5)	0.40095 (16)	0.0192 (6)	
C4	0.04586 (19)	1.1481 (6)	0.34526 (17)	0.0255 (6)	
H4	0.0848	1.2774	0.3268	0.031*	
C5	−0.0419 (2)	1.1495 (6)	0.31570 (18)	0.0299 (7)	
H5	−0.0623	1.2800	0.2775	0.036*	
C6	−0.09871 (18)	0.9637 (6)	0.34150 (17)	0.0236 (6)	
H6	−0.1583	0.9647	0.3212	0.028*	
C7	−0.06860 (19)	0.7743 (6)	0.39746 (19)	0.0301 (7)	
H7	−0.1076	0.6453	0.4160	0.036*	
C8	0.01881 (19)	0.7741 (6)	0.42627 (19)	0.0321 (7)	
H8	0.0393	0.6426	0.4642	0.039*	
C9	0.44368 (17)	0.9293 (6)	0.59946 (15)	0.0192 (5)	
H9	0.4991	0.9036	0.6280	0.023*	
C10	0.39017 (17)	1.1365 (5)	0.62925 (15)	0.0188 (5)	
C11	0.37208 (19)	1.4495 (6)	0.71860 (17)	0.0250 (6)	
H11	0.3819	1.5641	0.7637	0.030*	
C12	0.30047 (19)	1.4555 (6)	0.66655 (17)	0.0246 (6)	
H12	0.2518	1.5715	0.6685	0.030*	
C13	0.31160 (18)	1.2562 (5)	0.60850 (17)	0.0224 (6)	
H13	0.2721	1.2137	0.5637	0.027*	

### Atomic displacement parameters (Å^2^)

**Table:**

	*U*^11^	*U*^22^	*U*^33^	*U*^12^	*U*^13^	*U*^23^
Cu	0.0141 (2)	0.0252 (3)	0.0192 (3)	−0.00134 (18)	0.00266 (17)	−0.00100 (19)
S1	0.0163 (3)	0.0344 (4)	0.0207 (3)	0.0034 (3)	−0.0001 (3)	−0.0076 (3)
S2	0.0153 (3)	0.0256 (4)	0.0222 (3)	0.0000 (3)	−0.0016 (2)	−0.0025 (3)
O1	0.0217 (10)	0.0322 (11)	0.0178 (9)	0.0049 (8)	0.0005 (7)	−0.0047 (8)
N1	0.0126 (10)	0.0229 (12)	0.0202 (11)	0.0010 (9)	0.0013 (8)	0.0005 (9)
N2	0.0106 (10)	0.0216 (11)	0.0186 (10)	−0.0031 (8)	0.0027 (8)	0.0015 (9)
C1	0.0140 (12)	0.0192 (13)	0.0224 (13)	−0.0040 (10)	0.0020 (10)	0.0024 (11)
C2	0.0168 (13)	0.0239 (14)	0.0251 (14)	−0.0007 (11)	−0.0019 (10)	−0.0021 (11)
C3	0.0163 (12)	0.0223 (14)	0.0187 (13)	0.0023 (10)	−0.0002 (10)	−0.0046 (11)
C4	0.0253 (14)	0.0257 (15)	0.0251 (14)	−0.0018 (12)	−0.0008 (11)	0.0018 (12)
C5	0.0298 (16)	0.0312 (16)	0.0276 (15)	0.0017 (13)	−0.0052 (12)	0.0073 (13)
C6	0.0195 (13)	0.0282 (15)	0.0225 (14)	0.0060 (11)	−0.0018 (11)	−0.0057 (12)
C7	0.0193 (14)	0.0341 (17)	0.0366 (17)	−0.0021 (12)	0.0012 (12)	0.0086 (14)
C8	0.0232 (14)	0.0346 (17)	0.0376 (17)	0.0006 (13)	−0.0030 (12)	0.0166 (14)
C9	0.0137 (12)	0.0264 (14)	0.0176 (12)	−0.0014 (10)	0.0018 (10)	0.0006 (11)
C10	0.0184 (12)	0.0225 (14)	0.0158 (12)	−0.0050 (11)	0.0032 (10)	0.0014 (11)
C11	0.0291 (15)	0.0280 (15)	0.0184 (13)	0.0017 (12)	0.0052 (11)	−0.0007 (12)
C12	0.0231 (14)	0.0230 (14)	0.0280 (15)	0.0028 (11)	0.0045 (11)	−0.0004 (12)
C13	0.0211 (13)	0.0213 (14)	0.0245 (14)	0.0000 (11)	−0.0007 (11)	−0.0017 (11)

### Geometric parameters (Å, º)

**Table:**

Cu—S1	2.1845 (7)	C4—C5	1.397 (4)
Cu—N2	1.923 (2)	C4—H4	0.9500
Cu—N2^i^	1.923 (2)	C5—C6	1.369 (4)
Cu—S1^i^	2.1845 (7)	C5—H5	0.9500
C1—S1	1.720 (3)	C6—C7	1.386 (4)
C1—S2	1.753 (2)	C6—H6	0.9500
S2—C2	1.823 (3)	C7—C8	1.389 (4)
O1—C11	1.358 (3)	C7—H7	0.9500
O1—C10	1.384 (3)	C8—H8	0.9500
N1—N2	1.409 (3)	C9—C10	1.438 (4)
C1—N1	1.286 (3)	C9—H9	0.9500
C9—N2	1.300 (3)	C10—C13	1.367 (4)
C2—C3	1.511 (3)	C11—C12	1.346 (4)
C2—H2A	0.9900	C11—H11	0.9500
C2—H2B	0.9900	C12—C13	1.413 (4)
C3—C8	1.379 (4)	C12—H12	0.9500
C3—C4	1.378 (4)	C13—H13	0.9500
			
N2—Cu—N2^i^	180.00 (11)	C6—C5—C4	120.4 (3)
S1—Cu—N2	85.83 (6)	C6—C5—H5	119.8
N2^i^—Cu—S1	94.17 (6)	C4—C5—H5	119.8
S1^i^—Cu—N2	94.18 (6)	C5—C6—C7	119.5 (3)
N2^i^—Cu—S1^i^	85.82 (6)	C5—C6—H6	120.2
S1—Cu—S1^i^	180.0	C7—C6—H6	120.2
C1—S1—Cu	95.74 (9)	C6—C7—C8	119.7 (3)
C1—S2—C2	101.88 (12)	C6—C7—H7	120.2
C11—O1—C10	106.7 (2)	C8—C7—H7	120.2
C1—N1—N2	112.0 (2)	C3—C8—C7	121.3 (3)
C9—N2—N1	112.6 (2)	C3—C8—H8	119.4
C9—N2—Cu	126.72 (18)	C7—C8—H8	119.4
N1—N2—Cu	120.67 (16)	N2—C9—C10	128.2 (2)
S1—C1—S2	115.03 (15)	N2—C9—H9	115.9
S1—C1—N1	125.08 (19)	C10—C9—H9	115.9
S2—C1—N1	119.9 (2)	C13—C10—O1	108.9 (2)
C3—C2—S2	108.50 (18)	C13—C10—C9	138.3 (2)
C3—C2—H2A	110.0	O1—C10—C9	112.8 (2)
S2—C2—H2A	110.0	C12—C11—O1	110.7 (2)
C3—C2—H2B	110.0	C12—C11—H11	124.7
S2—C2—H2B	110.0	O1—C11—H11	124.7
H2A—C2—H2B	108.4	C11—C12—C13	106.9 (3)
C8—C3—C4	118.5 (3)	C11—C12—H12	126.6
C8—C3—C2	120.1 (2)	C13—C12—H12	126.6
C4—C3—C2	121.4 (3)	C10—C13—C12	106.8 (2)
C3—C4—C5	120.6 (3)	C10—C13—H13	126.6
C3—C4—H4	119.7	C12—C13—H13	126.6
C5—C4—H4	119.7		
			
C1—N1—N2—C9	173.3 (2)	C5—C6—C7—C8	−0.4 (5)
C1—N1—N2—Cu	−6.9 (3)	C4—C3—C8—C7	−0.7 (5)
N2—N1—C1—S1	0.0 (3)	C2—C3—C8—C7	179.9 (3)
N2—N1—C1—S2	179.76 (17)	C6—C7—C8—C3	0.7 (5)
Cu—S1—C1—N1	5.3 (2)	N1—N2—C9—C10	−0.8 (4)
Cu—S1—C1—S2	−174.52 (13)	Cu—N2—C9—C10	179.4 (2)
C2—S2—C1—N1	1.1 (3)	C11—O1—C10—C13	0.4 (3)
C2—S2—C1—S1	−179.07 (15)	C11—O1—C10—C9	179.1 (2)
C1—S2—C2—C3	−168.47 (19)	N2—C9—C10—C13	−4.3 (6)
S2—C2—C3—C8	82.6 (3)	N2—C9—C10—O1	177.5 (2)
S2—C2—C3—C4	−96.8 (3)	C10—O1—C11—C12	0.0 (3)
C8—C3—C4—C5	0.5 (4)	O1—C11—C12—C13	−0.4 (3)
C2—C3—C4—C5	179.9 (3)	O1—C10—C13—C12	−0.6 (3)
C3—C4—C5—C6	−0.2 (5)	C9—C10—C13—C12	−178.8 (3)
C4—C5—C6—C7	0.2 (5)	C11—C12—C13—C10	0.6 (3)

Symmetry code: (i) −*x*+1, −*y*+1, −*z*+1.

### Hydrogen-bond geometry (Å, º)

*Cg*1 is the centroid of the (C3–C8) ring.

**Table:**

*D*—H···*A*	*D*—H	H···*A*	*D*···*A*	*D*—H···*A*
C5—H5···*Cg*1^ii^	0.95	2.96	3.646 (3)	131

Symmetry code: (ii) −*x*, *y*+1/2, −*z*+1/2.
